# Bromodomain dimers: A case study of BRD4 and family-wide AlphaFold predictions

**DOI:** 10.1063/4.0001211

**Published:** 2026-05-22

**Authors:** Alisa S. Dengler, Lara Lunglmeir, Matthias J. Brandl, T. Reid Alderson

**Affiliations:** 1 Helmholtz Munich, Molecular Targets and Therapeutics Center, Institute of Structural Biology, 85764 Neuherberg, Germany; 2 Technical University of Munich, TUM School of Natural Sciences, Department of Bioscience, Bavarian NMR Center, 85747 Garching, Germany; 3 Helmholtz Munich, Computational Health Centre, Institute of Computational Biology, 85764 Neuherberg, Germany

## Abstract

Bromodomains are conserved acetyl-lysine reader domains that play a central role in the assembly of transcriptional regulatory complexes. While generally presumed to function as monomers, bromodomain homo-dimers have been identified, and several bromodomain-containing proteins have been linked to biomolecular condensates, where locally elevated concentrations may promote dimerization. Here, we investigated bromodomain dimerization with an integrated approach that combines structural and biophysical measurements with AlphaFold-based predictions across the bromodomain family. Using the second bromodomain (BD2) of BRD4 as a model system, we characterized the thermodynamics and kinetics of its monomer-dimer equilibrium by two-dimensional nuclear magnetic resonance (NMR) lineshape analysis and CPMG relaxation dispersion. We found that the BRD4^BD2^ dimer forms transiently with a dissociation constant near 400 μM and a lifetime near 1 ms. Using our NMR-derived restraints, we performed data-driven docking to generate models of the BRD4^BD2^ dimer. To assess dimerization propensity across the wider bromodomain family, we leveraged AlphaFold-Multimer and AlphaFold3 to systematically predict homo-dimeric models for all human bromodomains. We identified several predicted dimer architectures, with 15 bromodomain dimers that have higher interface-confidence scores than BRD4^BD2^. Overall, our results suggest that weak and reversible dimerization may be more widespread among bromodomains, where it could contribute to function in dynamic transcriptional assemblies.

## INTRODUCTION

The assembly of proteins into functional complexes underlies nearly all cellular processes.^1–3^ Most complexes are composed of identical units and form symmetric homo-oligomers,^4,5^ whose repetitive features can enable cooperativity, long-range allostery, and multi-valency.^4^ While some oligomers form permanently and irreversibly, others assemble transiently and in response to changes in cellular conditions.^3,6–9^ Transient oligomerization can modulate protein functions, subcellular localization, and interaction networks,^6–9^ with dynamic oligomers implicated in the onset of protein aggregation^10,11^ and formation of biomolecular condensates.^12,13^

Biomolecular condensates form through networks of weak, multivalent interactions including protein oligomerization.^14^ Many structured protein domains have been identified in condensate-forming proteins or directly implicated in condensate formation.^15–17^ Within condensates, locally increased protein concentrations, on the order of hundreds of mg/mL,^18,19^ may promote the oligomerization of protein domains that are otherwise primarily monomeric under dilute conditions. Moreover, the context dependence of protein oligomerization complicates the interpretation of oligomeric states that are inferred from static crystal structures or biophysical measurements that were performed at one protein concentration.^3,20^ For instance, a recent study of 17 homo-oligomers found that half of the proteins existed in oligomeric forms that differed from database annotations.^21^ Consistent with this, the second bromodomain of BRD4 was only recently reported to homo-dimerize^22^ despite extensive biophysical and structural studies.^23–29^ Moreover, the bromodomains in PCAF and GCN5 (PDB:^30^ 3gg3, 3d7c) both formed dimers in the crystal lattice but were not detected in solution.^31^

Bromodomains are often part of larger, multidomain proteins that are involved in the regulation of chromatin structure and transcription.^31,32^ A prominent subgroup is formed by the bromodomain and extra-terminal domain (BET) proteins, including BRD2, BRD3, BRD4, and BRDT, which contain two N-terminal bromodomains (BD1 and BD2) and a C-terminal extra-terminal domain.^33^ BET proteins function both as readers of acetylation marks and as molecular scaffolds for the assembly of transcriptional complexes.^33,34^ Within the BET family, BRD4 is a multi-functional protein^34,35^ that is closely associated with transcriptionally active chromatin and the formation of condensates at super-enhancers.^36–38^ These BRD4-containing condensates spatially concentrate the transcriptional machinery and support robust gene expression.^36^ BRD4 phase separation depends on its intrinsically disordered regions and tandem bromodomains (BDs): deletion of the BDs or inhibition of acetyl-lysine binding attenuates BRD4 condensation.^37,39^ Consistent with this, BD-mediated dimerization of BRD4 has been detected in cells upon binding to acetylated chromatin.^40^ Thus, BD dimerization could become functionally relevant within condensates where the concentration of BRD4 is highly enriched. More generally, this raises the possibility that BD self-assembly may play a role in the dense, multivalent networks of other transcriptional condensates. Including BRD4, more than ten bromodomain-containing proteins have been associated with biomolecular condensates.^16^

Here, we characterized the thermodynamics and kinetics of dimerization in the second bromodomain of BRD4 (BRD4^BD2^). Using an integrated approach that combines nuclear magnetic resonance (NMR) spectroscopy, small-angle x-ray scattering (SAXS), and NMR-guided structural modeling, we show that the BRD4^BD2^ dimer transiently forms with a lifetime near 1 ms and a dissociation constant near 400 μM. Our NMR-driven models of the BRD4^BD2^ dimer reveal a charged interface that involves the αB and αC helices. Extending beyond BRD4, we leverage AlphaFold-Multimer and AlphaFold3 to systematically predict the prevalence and architectures of dimerization across the human bromodomain family. Together, our results reveal bromodomain dimerization as an underappreciated and potentially widespread interaction mode that may become functionally relevant in transcriptional condensates.

## RESULTS

BRD4^BD2^ adopts the common bromodomain fold that comprises four α-helices (αZ, αA, αB, and αC). These helices are connected by two loop regions, the ZA and BC loops,^33^ which define the acetyl-lysine binding pocket^41,42^ that mediates the recruitment of chromatin-associated proteins.^31^ The structure, dynamics, and binding interactions of BRD4^BD2^ have been extensively studied by NMR spectroscopy^22–29^ in which the protein was generally presumed to be monomeric. However, Wernersson *et al.*^22^ recently identified weak dimerization of BRD4^BD2^ in both the unbound and peptide-bound forms of the domain. Here, we built upon this previous work to further characterize the dimerization of BRD4^BD2^ using NMR spectroscopy.

**FIG. 1.**
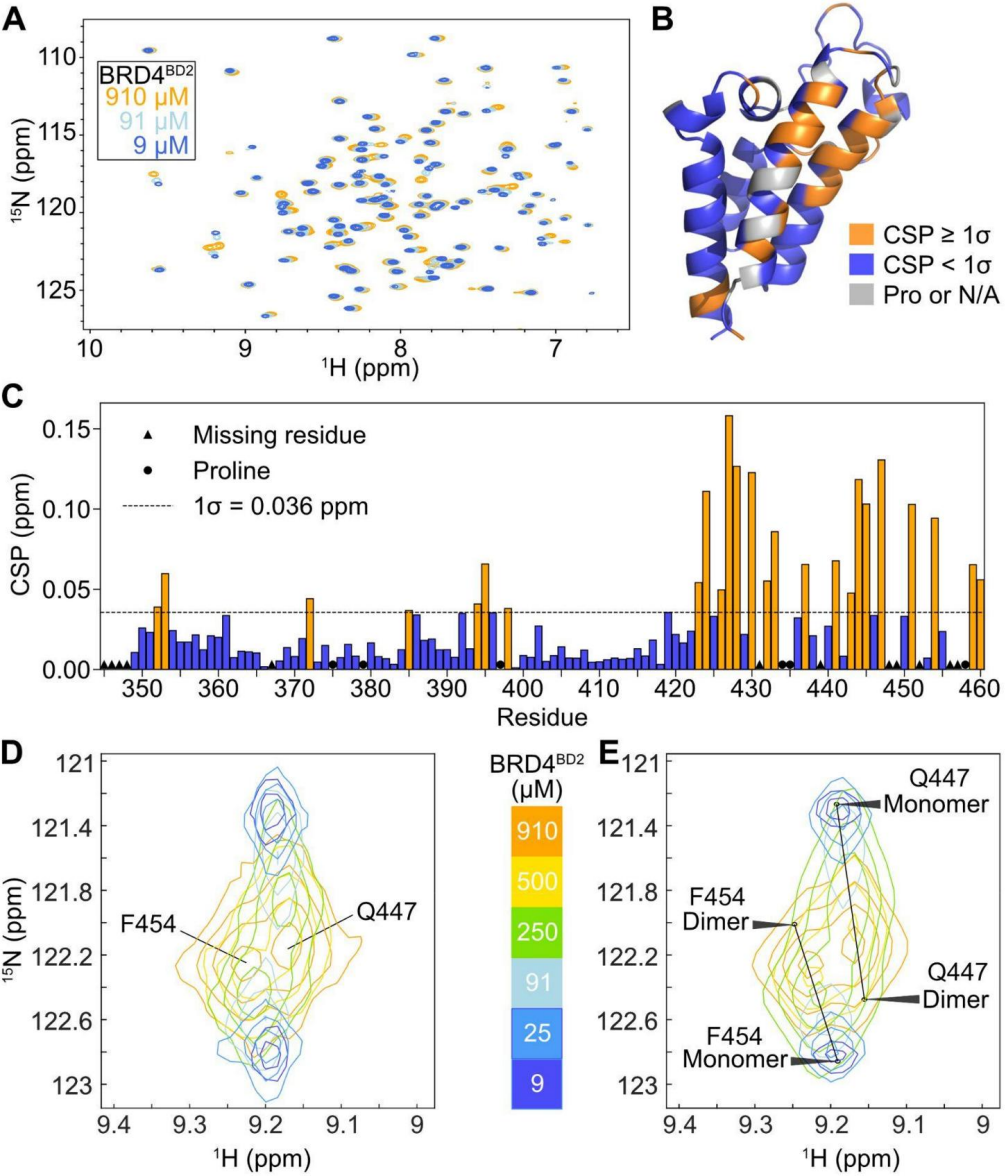
Dimerization of BRD4^BD2^ monitored by NMR spectroscopy. (a) 2D ^1^H–^15^N HSQC spectra of ^15^N-BRD4^BD2^ at 11.7 T at three different protein concentrations: 910 μM (orange), 91 μM (cyan), and 9 μM (blue). All solution conditions were otherwise identical (293 K, 25 mM MES, 50 mM NaCl, 0.5 mM EDTA, 1 mM TCEP, 0.02% sodium azide, pH 6.5). (b) Combined, weighted amide CSPs are shown on the structure of BRD4^BD2^ (PDB ID: 2ouo), with orange (blue) residues showing CSPs that are greater than or equal to (less than) one standard deviation among all residues. Grey residues indicate proline residues, which are not detected in this experiment, or otherwise missing or not assigned resonances. (c) The CSPs from panel B are shown on a continuous scale in per-residue format. The dashed line indicates one standard deviation, and proline residues as well as residues whose NMR signals are missing or unassigned are indicated. (d) and (e) Zoomed-in region from the NMR-TITAN fits of the BRD4^BD2^ titration data showing experimental (d) and simulated (e) spectra for each of the indicated protein concentrations. The NMR-TITAN-derived chemical shifts of the pure monomer and pure dimer are indicated in (e).

We recombinantly expressed and purified ^15^N-labeled human BRD4^BD2^, encompassing residues E345–E460 (full-length numbering). The construct includes a total of 116 residues and corresponds to a molecular mass of 13.5 kDa. The two-dimensional (2D) ^1^H-^15^N heteronuclear single-quantum coherence (HSQC) spectrum of ^15^N-labeled BRD4^BD2^ showed that our construct was folded and gave rise to the expected number of cross-peaks [Fig. 1(a)]. To enable mapping of residues in crowded spectral regions, we transferred BRD4^BD2^ resonance assignments from the Biological Magnetic Resonance Bank (BMRB)^43^ (entry 15057, 51417). Furthermore, we manually corroborated and extended these assignments by collecting non-uniformly sampled triple-resonance spectra on a ^13^C,^15^N-labeled sample of BRD4^BD2^ (Methods, supplementary material Fig. 1). Overall, we assigned 101 of 109 ^1^H^N^ (93%), 101 of 109 ^15^N (93%), 111 of 115 ^13^CO (97%), 111 of 115 ^13^CA (97%), and 93 of 112 ^13^CB (83%) resonances (Methods). Besides the N-terminal E345 and all proline residues, the missing ^1^H/^15^N assignments include K346 (i + 1 to the N-terminus), K404, K431, V439, D448, M452, K456, and M457. These sites are also not assigned in some BMRB entries for BRD4^BD2^ (e.g., 50146) and almost exclusively reside in the C-terminal αC helix, which forms a major part of the dimer interface (see below). We used TALOS-N^44^ to predict the backbone dihedral angles of BRD4^BD2^ based on the assigned ^1^H^N^, ^15^N, ^13^CO, ^13^CA, and ^13^CB chemical shifts. The TALOS-N-derived secondary structure of BRD4^BD2^ is largely consistent with the available structures of unligated BRD4^BD2^ (supplementary material Fig. 2). However, we note that the TALOS-N-predicted dihedral angles for loop residues A373-Y377 and Y390-I393, which are helical in the crystal structure of BRD4^BD2^, suggest that the short helices are only partially formed in solution (supplementary material Fig. 2).

### BRD4^BD2^ dimerization detected by NMR spectroscopy

BRD4^BD2^ was previously reported to dimerize,^22^ based on quantitative analysis of NMR chemical shifts and spin relaxation rates that were obtained at two different protein concentrations. We examined the BRD4^BD2^ monomer-dimer equilibrium in our construct, which spans E345-E460 and lacks the first four residues used previously.^22^ We recorded 2D ^1^H-^15^N HSQC spectra of ^15^N-labeled BRD4^BD2^ across a range of protein concentrations: 9, 25, 50, 91, 200, 500, and 910 μM [Fig. 1(a)]. To reduce the contributions from chemical exchange-induced broadening (see below), we collected NMR spectra at a static magnetic field strength of 11.7 T (500 MHz ^1^H Larmor frequency).

For a number of cross-peaks in the spectrum of BRD4^BD2^, we observed progressive changes in their chemical shifts as the protein concentration increased [Fig. 1(a)]. This is consistent with previous observations,^22^ despite slightly different construct boundaries (H341-E460 vs E345-E460). We calculated the combined, weighted amide chemical shift perturbations (CSPs) (Methods) between the spectra at 9 and 910 μM [Figs. 1(b) and 1(c)] and plotted these CSPs on the structure of monomeric, unligated BRD4^BD2^ (PDB: 2ouo). Residues exhibiting CSPs greater than or equal to 0.05 ppm include Q353, K395, R423, L424, S427, N428, Y430, Y432, N433, H437, A441, R444, K445, Q447, E451, F454, D459, and E460. These residues are likely situated at or near the dimer interface [Figs. 1(b) and 1(c)]. Many of our unassigned ^1^H-^15^N signals are close to these residues (K431, V439, D448, M452, K456, and M457), suggesting that the unassigned cross-peaks may be broadened beyond detection by contributions from monomer-dimer exchange. Previously, a comparison of NMR spectra between more similar BRD4^BD2^ concentrations (526 and 120 μM) identified fewer CSPs that were greater than or equal to 0.05 ppm, including L424, Y430, K445, Q447, E451, and E460. These residues overlap with our results [Figs. 1(b) and 1(c)], which are expanded due to the larger magnitude of the CSPs that manifest through the greater difference in molar fractions of monomer and dimer. Overall, our results confirm the monomer-dimer equilibrium of BRD4^BD2^, building upon the previously reported results,^22^ and indicate that helices αB and αC comprise the dimer interface.

Previously, a high-resolution solution structure of monomeric BRD4^BD2^ was determined in which the construct included a C-terminal hexa-histidine (His_6_) tag that was not removed prior to NMR data collection.^25^ The protein reportedly remained monomeric at a protein concentration of 1 mM at pH 6. Under these conditions, the His_6_ tag would be expected to be significantly protonated, which could have prevented dimer formation via charge-charge repulsion if the two His_6_ tags were in close proximity in the dimer. Therefore, we tested if a C-terminal His_6_ tag on BRD4^BD2^ affects dimer formation. To this end, we compared 2D ^1^H-^15^N HSQC spectra of ^15^N-labeled BRD4^BD2^ with and without the C-terminal His_6_ tag at pH 6.5 (supplementary material Fig. 3). The spectrum of the His_6_-tagged BRD4^BD2^ sample was diagnostic of BD2 dimerization^22^ (supplementary material Fig. 3). Thus, we concluded that the C-terminal His_6_ tag does not impact BRD4^BD2^ dimerization, and we used the untagged form of the protein in all experiments henceforth.

### Kinetics and thermodynamics of BRD4^BD2^ dimerization from 2D NMR lineshape analysis

To extract additional kinetic and thermodynamic insight into the self-association of BRD4^BD2^, we quantitatively analyzed our NMR titration data with the 2D lineshape analysis method, NMR titration analysis (TITAN)^45^ [Figs. 1(d) and 1(e), supplementary material Fig. 4]. Whereas NMR titration analyses that fit CSPs or intensities are limited to resonances that are respectively in the fast or slow exchange regime,^46^ NMR-TITAN can model all exchange regimes simultaneously. By numerically simulating the evolution of magnetization during a pulse sequence in the presence of chemical exchange, NMR-TITAN minimizes the difference between simulated and experimental 2D NMR spectra for a given binding reaction and set of NMR, kinetic, and thermodynamic parameters.^45^

Depending on the BRD4^BD2^ cross-peak of interest, and at a static magnetic field strength of 11.7 T, the exchange regime falls in the fast-to-intermediate regime on the chemical shift timescale [Fig. 1(a)]. From the largest observable ^15^N chemical shift difference of ca. 1 ppm (ca. 315 rad s^−1^ at 11.7 T), we can estimate that the exchange rate, *k*_ex_, must therefore be faster than 315 s^−1^. For a monomer-dimer equilibrium, *k*_ex_ is given by *k*_ex_ = 2*k*_on_[M]_free_ + *k*_off_, where [M]_free_ corresponds to the concentration of free monomer.^47^ If we assume a diffusion-limited^48^ association rate of 10^5^-10^6^ M^−1^ s^−1^, then *k*_ex_ is dominated by the dissociation rate (*k*_off_) when [M]_free_ is limiting (i.e., *k*_ex_ ≈ *k*_off_). Therefore, we can estimate from our titration series that the BRD4^BD2^ dimer dissociation rate, *k*_off_, must be greater than or equal to 315 s^−1^.

We fit our BRD4^BD2^ titration data to a homo-dimerization model that is described globally by the dissociation rate constant (*k*_off_), association rate constant (*k*_on_), and dissociation constant (*K*_d_) with the internal relation *K*_d_ = *k*_off_/*k*_on_. For a given *K*_d_ and total protein concentration, the concentration of monomer, and thus dimer, can be analytically calculated (Methods). For each state (monomer, dimer), the locally fitted NMR parameters include per-residue ^1^H and ^15^N chemical shifts and relaxation rates (supplementary material Table 1). With a global fit of 18 residues (supplementary material Fig. 4), NMR-TITAN returns a *K*_d_ of 391 ± 12 μM with a *k*_off_ of 944 ± 65 s^−1^ and *k*_on_ of 2.41 ± 0.18 × 10^6^ M^−1^ s^−1^ where the errors are derived from a bootstrap analysis [Figs. 1(d) and 1(e), Table I, supplementary material Table 1]. Our fitted *K*_d_ closely agrees with the value that was derived previously^22^ (350 ± 90 *μ*M) based on quantitative considerations of NMR peak intensities at two different BRD4^BD2^ concentrations.

**TABLE I.** Thermodynamic and kinetic parameters of BRD4^BD2^ dimerization. The fitted parameters were derived from a 2D lineshape analysis of NMR spectra that were collected at 11.7 T in 25 mM MES, 50 mM NaCl, 0.5 mM EDTA, and 1 mM TCEP at pH 6.5 and 293 K.

Parameter	Value
*K*_d_	391 ± 12 μM
*k*_off_	944 ± 65 s^−1^
*k*_on_	2.41 ± 0.18 × 10^6^ M^−1^ s^−1^

From our fitted values, and with knowledge of the total protein concentration, we can compute^47^ the rate of interconversion between monomer and dimer, *k*_ex_ = 2*k*_on_[M] + *k*_off_. Using the NMR-TITAN-fit parameters at a total protein concentration of 910 μM, corresponding to a free monomer concentration of 335 μM, the calculated *k*_ex_ is then approximately 2500 s^−1^. Upon decreasing the total protein concentration to 9 μM, *k*_ex_ correspondingly decreases to 980 s^−1^. Indeed, given this value of *k*_ex_, most ^15^N chemical shift differences map to the fast exchange regime at 11.7 T (*k*_ex_ > |Δω|), whereas several resonances with larger ^1^H chemical shift differences approach the intermediate exchange regime. NMR-TITAN quantitatively models the lineshapes across the BRD4^BD2^ titration series, yielding high-quality fits in which intensity is quantitatively recovered (supplementary material Fig. 5).

### Characterizing BRD4^BD2^ dimerization by CPMG relaxation dispersion

The fitted kinetic parameters from our lineshape analysis [Figs. 1(d) and 1(e), Table I, supplementary material Table 1] indicate that BRD4^BD2^ interconverts between monomeric and dimeric forms on the micro-to-millisecond timescale. Carr-Purcell-Meiboom-Gill (CPMG) relaxation dispersion is a powerful NMR experiment that can quantify the interconversion on the micro-to-millisecond timescale between an NMR-observable state and an otherwise “NMR-invisible” state that may be fractionally populated as low as 0.5%.^49,50^ In this experiment, the effective *R*_2_ rate (*R*_2,eff_) is measured as a function of the number of 180° refocusing pulses that are applied during a constant-time relaxation delay.^51,52^ Variations in *R*_2,eff_ are indicative of micro-to-millisecond timescale exchange between two or more states with a non-zero chemical shift difference between the interconverting states.^53^ Quantitative analysis of the dispersion data provides kinetic (interconversion rate, *k*_ex_ = *k*_AB_ + *k*_BA_), thermodynamic (populations, *p*_A_ + *p*_B_ = 1), and structural insight (chemical shift difference, | Δω| = | ω_A_ − ω_B_|). CPMG relaxation dispersion has been used previously to characterize a variety of interconverting monomer-dimer systems.^10,11,54–58^

**FIG. 2.**
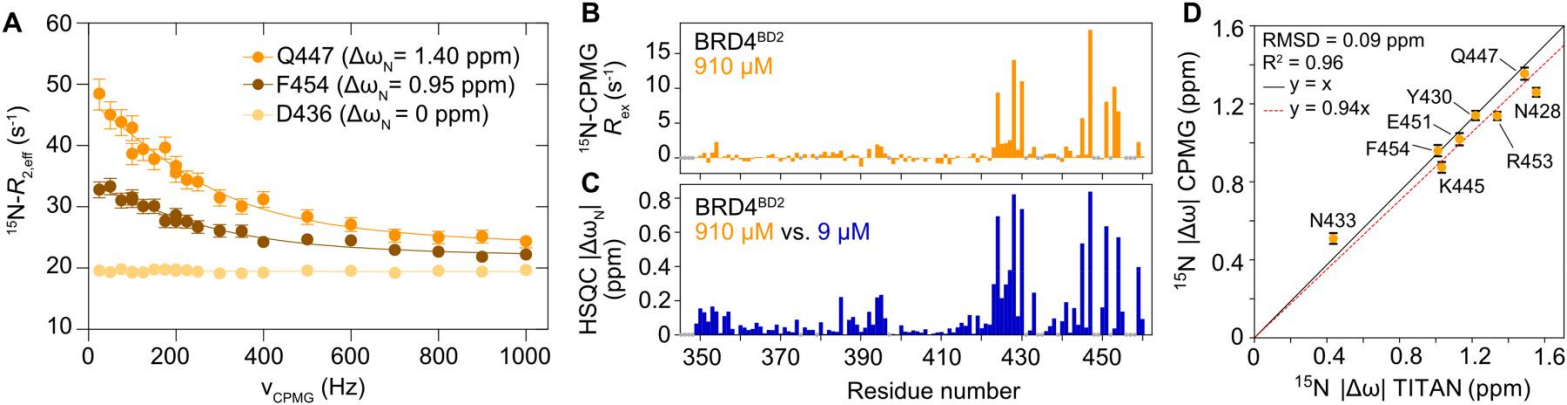
BRD4^BD2^ monomer-dimer interconversion detected by ^15^N-CPMG relaxation dispersion. (a) ^15^N-CPMG relaxation dispersion data for selected residues (Q447, F454, D436) at a total BRD4^BD2^ concentration of 910 μM. The ^15^N | Δω| values (dimer-monomer) derived from the TITAN 2D lineshape analysis are shown in parentheses, and the solid-lines represent a global fit to a model of two-site chemical exchange in which the fraction of monomer was fixed at 0.34 based on the known *K*_d_ and total protein concentration. (b) *R*_ex_ values derived from the ^15^N-CPMG relaxation dispersion data as a function of residue number. (c) Absolute value of the ^15^N chemical shift differences determined through comparison of 2D ^1^H-^15^N HSQC spectra at 910 and 9 μM (HSQC | Δω|_N_) shown as a function of residue number. Note that the TITAN and CPMG | Δω|_N_ values derive from pure monomer and dimer, whereas the HSQC | Δω|_N_ values are smaller and reflect the fractional population of the BRD4^BD2^ dimer. (d) Correlation between ^15^N |Δω| values derived from TITAN (x-axis) and CPMG relaxation dispersion (y-axis). The solid black line indicates y = x, and the red dashed line shows the best-fit line to *y* = *Mx* with *M* = 0.94. The R^2^ value is 0.96 (Pearson correlation) and the root mean square deviation is 0.09 ppm.

We recorded ^15^N-CPMG relaxation dispersion on a sample of ^15^N-labeled BRD4^BD2^ at 0.91 mM using a modified form of the ^15^N ST-CW-CPMG pulse sequence [Fig. 2(a)].^52,59^ A total of 13 residues yielded ^15^N dispersions with *R*_ex_ values greater than 2 s^−1^ [Fig. 2(b)], where *R*_ex_ is defined as the difference between *R*_2,eff_ values at low and high CPMG pulse repetition rates (Methods). These residues include L354, L424, M425, F426, S427, N428, Y430, K445, Q447, E451, R453, F454, and D459 [Fig. 2(b)]. These dispersions confirm that BRD4^BD2^ interconverts on the micro-to-millisecond timescale between a low-energy, ground-state conformation and an otherwise invisible, higher-energy state that is lowly populated. We identified that the minor state corresponds to the BRD4^BD2^ monomer in two different ways. First, we observed a correlation between the ^15^N CPMG-derived *R*_ex_ values measured in the 910 μM sample [Fig. 2(b)] and the HSQC-derived ^15^N |Δω| values that were obtained through direct comparison of ^15^N chemical shifts at 910 and 9 μM [Fig. 2(c)]. Second, we identified that the CPMG-detected chemical exchange process is concentration-dependent: increasing the total protein concentration to 1.6 mM decreased the values of *R*_ex_ (supplementary material Fig. 6), indicating that the population of the minor state decreases with total protein concentration. Taken together, these ^15^N CPMG relaxation dispersion results are consistent with a monomer-dimer exchange process on the micro-to-millisecond timescale in which we directly observed cross-peaks from the dimeric conformation while the monomer remained “invisible.” However, the presence of the monomer is amplified and relayed through chemical exchange with the directly detected dimer.

Since we determined the BRD4^BD2^ dimerization *K*_d_ under the same conditions where we collected ^15^N-CPMG relaxation dispersion data, we used the known protein concentration and *K*_d_ to calculate the expected percentage of BRD4^BD2^ monomer (34% at 0.91 mM *P*_t_ with *K*_d_ = 391 μM). We then fit our CPMG relaxation data with the value of *p*_B_ fixed at 34%, while all other exchange parameters were left free in the fit (Methods), in a manner similar to previously described approaches that leverage known dissociation constants.^60,61^ This yielded a best-fit *k*_ex_ value of 1692 ± 49 s^−1^ [Fig. 2(a)]. These values correspond to *k*_off_ and *k*_on_ values of 575 s^−1^ and 1.8 × 10^6^ M^−1^ s^−1^, respectively, which closely align with the obtained kinetic values from our independent 2D lineshape analysis (*k*_off_ = 944 ± 65 s^−1^, *k*_on_ = 2.42 ± 0.18 × 10^6^ M^−1^ s^−1^). The CPMG-derived *k*_off_ value indicates that the BRD4^BD2^ dimer has a lifetime (1/*k*_off_) of approximately 1.7 ms and is thus only transiently formed. Moreover, the best-fit values of ^15^N | Δω| for Q447 and F454 were 1.35 ± 0.03 and 0.96 ± 0.03 ppm [Fig. 2(a)], respectively, which agree well with the observed ^15^N |Δω| between the pure monomer and dimer (1.40 and 0.95 ppm, respectively, via NMR-TITAN) (supplementary material Table 1). Furthermore, the overall correlation between ^15^N-CPMG relaxation dispersion- and TITAN-fitted values of ^15^N | Δω| is evident from Fig. 2(d), with a root mean square deviation (RMSD) of 0.09 ppm and a Pearson correlation coefficient squared of 0.96. Note that the HSQC-derived ^15^N chemical shift differences under-estimate the magnitude of the ^15^N chemical shift change, as the 0.91 mM spectrum reflects a mixture of states in which the dimer is approximately only 66% populated [Fig. 2(c)]. By contrast, the ^15^N-CPMG relaxation dispersion experiment performed at a single protein concentration can extract the ^15^N chemical shift difference between the monomer and dimer in a similar manner as the 2D lineshape analysis.

### Hydrodynamic properties of the BRD4^BD2^ dimer from ^15^N relaxation, PFG-NMR, and SAXS

**FIG. 3.**
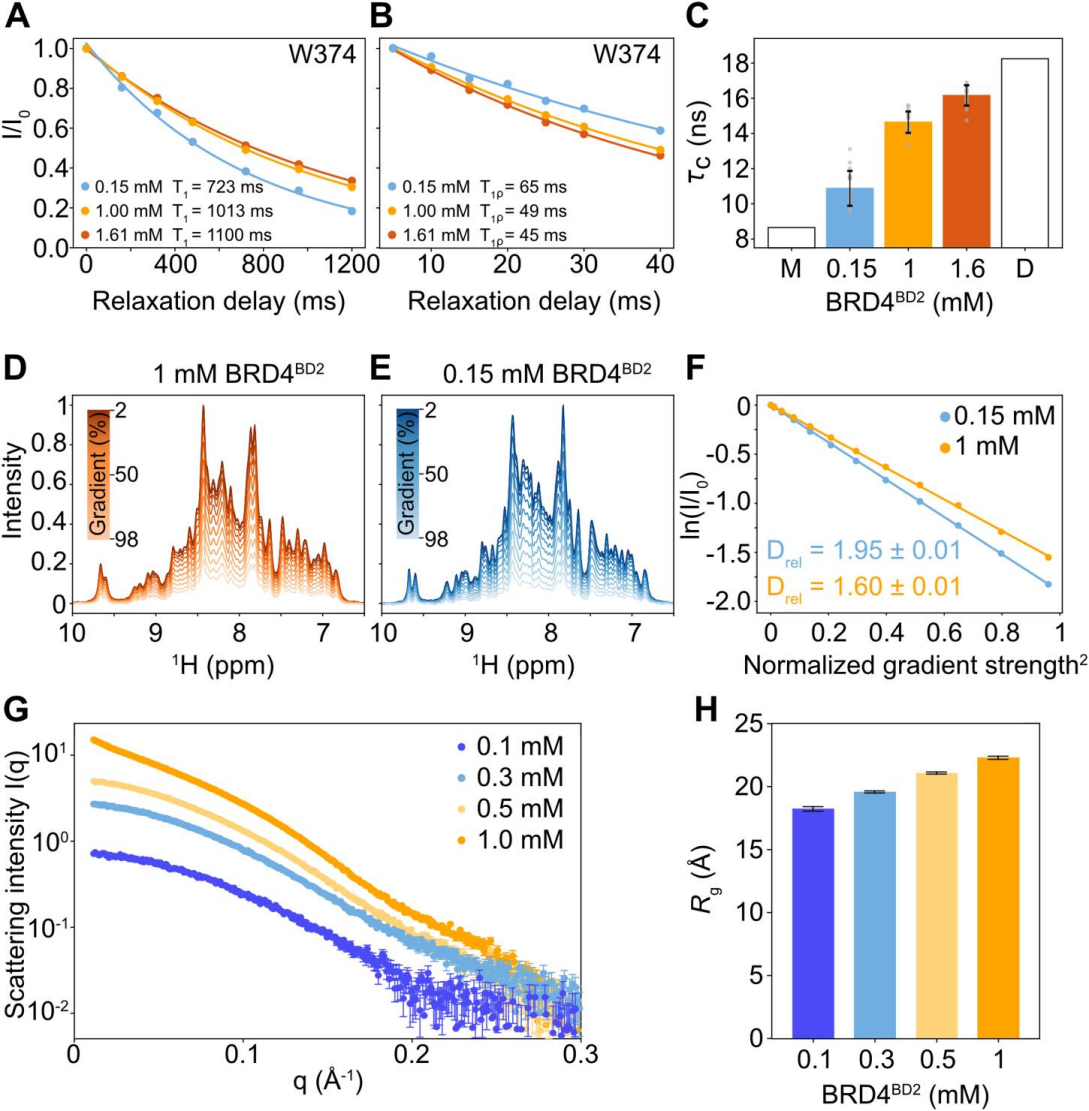
Hydrodynamic characterization of BRD4^BD2^ dimerization. ^15^N longitudinal (^15^N-*R*_1_) (a) and rotating frame (^15^N-*R*_1ρ_) relaxation (b) decay curves for W374 at three different BRD4^BD2^ concentrations (0.15, 1, 1.6 mM). The normalized intensities (I/I_0_) are shown and solid lines indicate monoexponential fits. (c) The rotational correlation time (τ_c_) of BRD4^BD2^, calculated from the measured ^15^N-*R*_1_ and ^15^N-*R*_2_ relaxation rates as a function of protein concentration. Predicted τ_c_ values for purely monomeric (M, PDB: 2ouo) and dimeric states of BRD4^BD2^ (D, AlphaFold3 prediction) are shown as reference. (d) and (e) PFG-NMR translational diffusion of BRD4^BD2^ at 1 mM (d) and 0.15 mM (e) total protein concentration with 12 spectra shown as a function of gradient strength. (f) The intensity decay [ln(I/I_0_)] is plotted as a function of the squared normalized gradient strength, and the fitted relative diffusion constants (*D*_rel_) are shown. The decreasing slope at higher concentration reflects reduced translational diffusion due to an increase in viscosity and an increased population of the dimer. (g) SAXS scattering profiles of BRD4^BD2^ at four protein concentrations (0.1, 0.3, 0.5, 1.0 mM). (h) SAXS-derived radius of gyration (R_g_) values from a Guinier analysis shown as a function of BRD4^BD2^ concentration.

To characterize the hydrodynamic properties of the BRD4^BD2^ monomer and dimer in solution, we recorded ^15^N spin relaxation, pulsed-field gradient (PFG)-NMR translational diffusion, and SAXSdata as a function of total protein concentration (Fig. 3). For an N-H bond vector within a macromolecule, the ratio of ^15^N longitudinal and transverse relaxation rates depends on the rotational correlation time,^62^ which is related to the shape and size of a macromolecule and provides a sensitive probe of the oligomeric state,^63^ including monomer-dimer equilibria.^64,65^

To this end, we first measured the ^15^N longitudinal (*R*_1_) and rotating-frame (*R*_1ρ_) relaxation rates in BRD4^BD2^ under otherwise identical conditions at three different protein concentrations: 150, 1000, and 1600 μM [Figs. 3(a) and 3(b)].^62,66–68^ After correcting our *R*_1ρ_ rates for ^15^N offset and ^15^N spin-lock field strength (2 kHz) to yield the *R*_2_ rates (Methods), we used the *R*_1_ and *R*_2_ rates to determine the rotational correlation time ( $\tau_{c}$) of BRD4^BD2^ as a function of total protein concentration [Fig. 3(c)]. This calculation (Methods) assumes isotropic rotational diffusion, which is an oversimplification of the axially symmetric rotational diffusion tensor that has been previously determined for BRD4^BD2^;^22^ however, the observed concentration-dependent changes in relaxation rates indicate that $\tau_{c}$ increases with concentration [Fig. 3(c)], which is indicative of a self-assembly process. At 150 μM protein concentration, the $\tau_{c}$ of BRD4^BD2^ was determined to be 10.9 ± 1 ns, which further increased to 14.6 ± 0.6 and 16.2 ± 0.6 ns at 1000 and 1600 μM, respectively [Fig. 3(c)]. HydroNMR^69^ simulations using the PDB structure of monomeric BRD4^BD2^ or an AlphaFold3-derived model of dimeric BRD4^BD2^ indicate theoretical $\tau_{c}$ values of 8.7 and 18.3 ns, respectively, which put lower and upper limits on the expected range of values [Fig. 3(c)]. With an increase in protein concentration, there will also be restricted rotational diffusion due to a small increase in solvent viscosity. However, we note that the observed changes here are larger than contributions from viscosity increase alone (see below).

Next, we collected NMR-based translational diffusion [Figs. 3(d)–3(f)] and SAXS measurements [Figs. 3(g) and 3(h)], which respectively provide access to hydration and gyration radii. In PFG-NMR translational diffusion, a gradient encodes the spatial location of a spin, and translational diffusion during a fixed delay time leads to incomplete rephasing upon gradient decoding. More specifically, the amount of signal attenuation depends on the translational diffusion coefficient, the strength and length of the encoding and decoding gradients, and the length of the delay time, as described by the Stejskal-Tanner equation (Methods). Thus, a molecule with a smaller radius of hydration (i.e., faster translational diffusion coefficient) will experience more signal attenuation than a particle with a larger radius of hydration, given otherwise identical gradient strengths and delays.

To compare the relative translational diffusion of BRD4^BD2^ at different protein concentrations, we collected 1D ^15^N-edited ^1^H spectra in which the strength of the encoding and decoding gradients was varied during a fixed diffusion delay time of 200 ms. Under the experimental conditions here (pH 6.5, 298 K), there will be hydrogen exchange between exposed amides and water during the fixed diffusion delay time, which may increase the fitted translational diffusion constant and differ between the monomer and dimer. However, the extent of protection from hydrogen exchange in the dimer is expected to be relatively small, given the rapid rate of monomer-dimer interconversion and the short lifetime of the dimer.

We obtained the intensity decay profiles at 150 and 1000 μM BRD4^BD2^ as shown in Figs. 3(d) and 3(e), respectively, and observed a decrease in the relative diffusion coefficient at the higher protein concentration [Fig. 3(f)]. The theoretical expectation for an increase in hydration radius upon dimerization^70^ is approximately 20% (Methods). The percent change in diffusion that we measured upon increasing the BRD4^BD2^ concentration from 150 to 1000 μM (21.8%) is consistent with a monomer-to-dimer transition. However, we note that both monomer and dimer are present in significant populations in both of these samples and interconvert during the diffusion delay. Therefore, the decreased diffusion at higher BRD4^BD2^ concentration likely arises from two contributions: an increased fraction of dimeric BRD4^BD2^ and an increase in solvent viscosity. Indeed, a comparison of the diffusion of a buffer component (EDTA) at 150 vs 1000 μM BRD4^BD2^ concentration indicates that the increase in viscosity is near 10%.

Next, we used SAXS as an orthogonal method to characterize the dimerization of BRD4^BD2^ in solution. The global, low-resolution information about particle shape and size derived from SAXS is highly complementary to local, atomic-level details from NMR.^71–76^ Moreover, SAXS data that are recorded as a function of protein concentration can be quantitatively modeled to describe oligomeric equilibria in solution.^77–79^ This is because the observed SAXS signal reflects the equilibrium mixture of states that are present in solution and depends both on the overall molecular mass (i.e., the number of protomers)^80^ and the topology or arrangement of protomers within an oligomer.^81^ To this end, we recorded SAXS data at four different BRD4^BD2^ concentrations (0.1, 0.3, 0.5, 1 mM) [Fig. 3(g)]. Particle aggregation was evident in the 1 mM scattering curve [Fig. 3(g)]. Nonetheless, qualitative interpretation of the fitted radii of gyration shows a progressive increase with BRD4^BD2^ concentration [Fig. 3(h)], which is expected for a monomer-dimer equilibrium. Furthermore, modeling of the molar fractions of monomeric and dimeric states at each concentration yielded values that are qualitatively consistent with our *K*_d_ value determined by NMR-TITAN (supplementary material Fig. 7).

### Structural modeling of the BRD4^BD2^ dimer

**FIG. 4.**
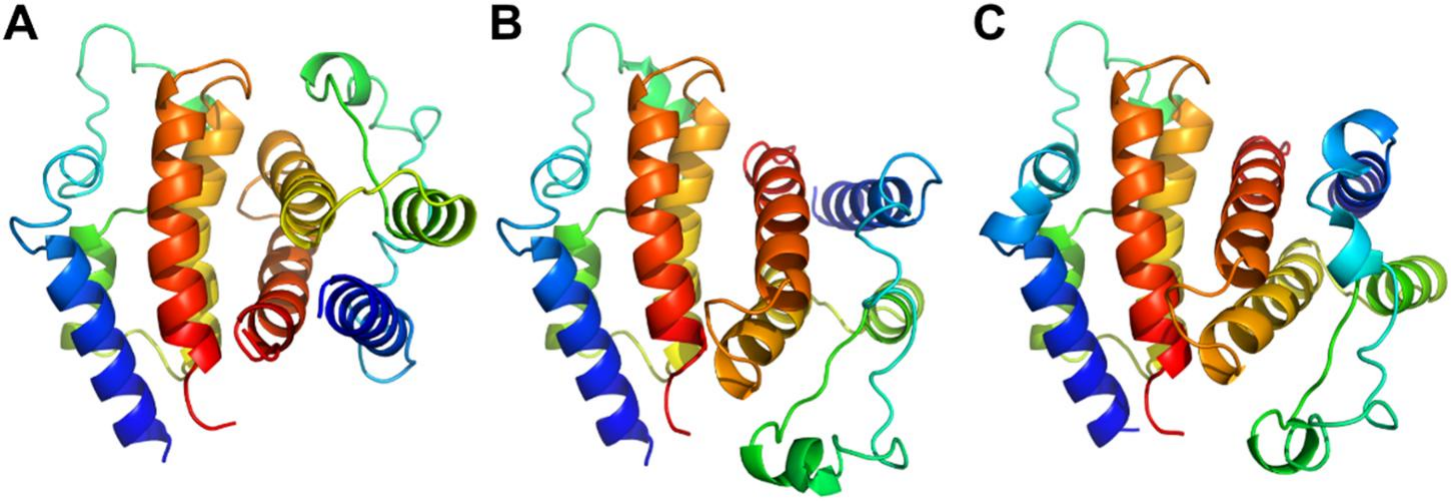
NMR-driven structural modeling of the BRD4^BD2^ dimer and comparison to AlphaFold3. (a) Structural model from the best-ranked (a) and second-best (b) HADDOCK cluster of a BRD4^BD2^ homo-dimer showing a symmetric dimer in which the interaction surface is primarily formed by helices αB and αC. (c) Top-ranked AlphaFold3 prediction of a BRD4^BD2^ homo-dimer from five random seeds. The predicted dimer architecture closely resembles the HADDOCK model in (b). All models are color-coded according to sequence position (N-terminus blue, C-terminus red).

To gain structural insight into the arrangement of the BRD4^BD2^ dimer, we performed an NMR-driven docking approach using our CSPs as input to the high-ambiguity data-driven docking (HADDOCK^82^) platform [Fig. 4(a)]. The top-scoring HADDOCK cluster yields a dimeric complex with a buried surface area (BSA) of 1120 Å^2^ per subunit involving 30 residues and 120 atoms. The total BSA of 2240 Å^2^ is comparable to previously reported values^3^ for weak homo-dimers (1670 ± 620 Å^2^). The residue content of the BRD4^BD2^ dimer interface is nearly equally distributed among groups, with 30% polar, 37% hydrophobic, and 33% charged residues (Methods). However, when evaluated by the percentage of the BSA, charged residues contribute most significantly with 49% of the total BSA followed by hydrophobic (27%) and polar residues (24%). The electrostatic surface of BRD4^BD2^ reveals a negatively charged patch near the N-terminus of αB and C-terminus of αC, whereas a positively charged region is located near the C-terminus of αB and N-terminus of αC (supplementary material Fig. 8). Furthermore, our NMR data were collected in buffer that contained 50 mM NaCl; thus, we tested if increasing the concentration of NaCl toward a more physiological range impacted BRD4^BD2^ dimer formation. The 2D ^1^H-^15^N HSQC spectrum of 0.5 mM ^15^N BRD4^BD2^ was nearly identical with 50 or 150 mM NaCl (supplementary material Fig. 9), indicating that the added NaCl did not disrupt dimer formation.

Thus, our NMR-driven HADDOCK model of the BRD4^BD2^ dimer reveals a primarily electrostatic interface involving favorable ionic contacts that form across the dimer interface (e.g., K395-D436, R423-D448, K456-D459). Among BDs in other BET family proteins, K395 is conserved in BD1 and BD2, whereas D436 is only conserved in BD2s and typically G or T in BD1s.^83^ We note similar trends for the R423-D448 and K456-D459 positions: these sites are conserved in BD2s but respectively replaced by N-K and [E/Q/S]-[I/T/Q] in BD1s.

We noticed that one protomer within our second-ranked HADDOCK cluster was rotated by approximately 180° relative to our top-scoring cluster [Fig. 4(b)]. This likely arises due to the lack of distance restraints in our calculation, and the ease of satisfying the observed CSPs through a simple symmetry rotation of one protomer. However, this prompted us to compare our HADDOCK models to a dimeric BRD4^BD2^ conformation predicted by AlphaFold3^84^ [Figs. 4(b) and 4(c)]. AlphaFold3 predicted a similar dimeric conformation to this HADDOCK cluster [Fig. 4(c)] with a backboneRMSD of 1.5 Å. By contrast, the RMSD of the AlphaFold3 dimer relative to the top-scoring HADDOCK cluster is 8.9 Å. Moreover, the overall topology of the AlphaFold3-predicted dimer differs from the crystal structure of the related BRD2^BD1^ (PDB: 1x0j), as the TM-score^85^ is approximately 0.6 for the dimer. The AlphaFold3-predicted model of the BRD4^BD2^ dimer, thus, involves similar interfacial residues that we detected here by NMR spectroscopy; however, we cannot determine the inter-protomer orientations with our experimental data. Nonetheless, the close resemblance between the experimental data-driven HADDOCK models and the AlphaFold3 computational prediction encouraged us to further pursue AlphaFold predictions with other bromodomain dimers below.

### AlphaFold predictions of human bromodomain dimers

Given our and previous observations of BRD4^BD2^ dimerization,^22^ we wondered if other bromodomains may dimerize in a similar manner. We first tested whether AlphaFold3 can reliably predict homo-dimeric models with a full-length bromodomain-containing protein, as exemplified by the 1362-residue BRD4 (supplementary material Fig. 10). A dimer of BRD4 totals 2724 residues of which more than 80% are predicted to be intrinsically disordered by the program AIUpred.^86^ We inferred four different homo-dimers of BRD4 by varying the random seed and compared the orientations of BD1 and BD2 (supplementary material Fig. 10). In three of the four seeds, we identified intra-molecular BD1-BD2 interactions that formed in both BRD4 chains. In one seed, however, we noticed an inter-molecular BD2-BD2 interaction with a similar inter-protomer orientation as observed in Fig. 4(c) with the isolated BD2 homo-dimer. The primary dimer interface in all models of full-length BRD4 involved the long helices that are predicted near the C-terminus of the protein; however, these C-terminal helices are associated with high predicted aligned error (PAE) values and low predicted local difference distance test (pLDDT) scores, suggesting that AlphaFold3 is not confident in their relative positions or conformations.

**FIG. 5.**
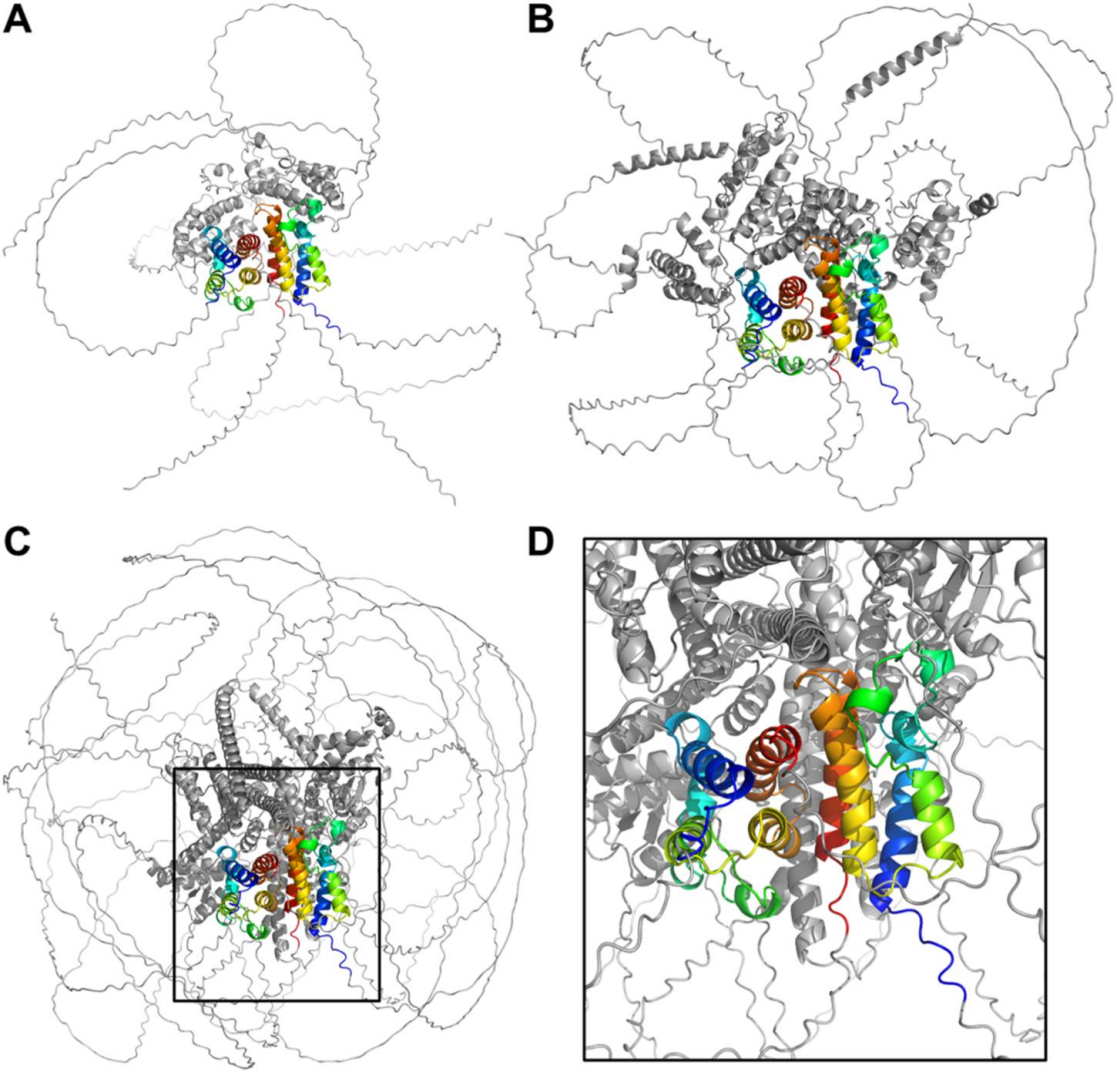
AlphaFold3 homo-dimer predictions for full-length BRD4 and truncations that lack the C-terminal intrinsically disordered region. AlphaFold3-predicted homo-dimer of BRD4 (a) residues 1-500 encompassing BD1, BD2, and the intervening linker; (b) residues 1-700 with the ET domain after BD2; and (c) residues 1-1362 (full-length), which contains a long C-terminal IDR after the ET domain. The black rectangle in (c) is shown in a zoomed-in view in panel (d). In all panels, the BD2 domain is shown in rainbow coloring from blue (N-terminus) to red (C-terminus) for clarity as well to identify its inter-domain orientation. Note that the AlphaFold3 predictions for full-length BRD4 are seed-dependent in their arrangement of BD1 and BD2 (see supplementary material Fig. 10).

Given the high inter-chain PAE values that indicate low confidence in the relative inter-domain orientations of the full-length BRD4 homo-dimer, we hypothesized that truncating the BRD4 sequence may improve the confidence metrics (Fig. 5). Indeed, recent work has shown that intrinsically disordered regions (IDRs) that are not involved in an intermolecular interface can lower the interface predicted template modeling (ipTM) score.^87,88^ Thus, we removed the C-terminal IDR (residues 701-1362) from the predictions and inferred homo-dimeric models for two different fragments that either contained BD1 and BD2 separated by the intervening linker [residues 1-500, Fig. 5(a)] or additionally contained the extra-terminal (ET) domain [residues 1-700, Fig. 5(b)]. In both cases, the BRD4 homo-dimeric models with IDR-truncated fragments formed inter-molecular BD2 interactions that were similarly identified in the isolated BRD4^BD2^ [Fig. 4(c)] as well as full-length BRD4 in a seed-dependent manner [Fig. 5(c), supplementary material Fig. 10]. Thus, a similar BD2 dimer interface and orientation is observed in these longer BRD4 constructs as with the isolated BRD4^BD2^ domain. However, the ipTM scores among BRD4 homo-dimers were consistently highest with the isolated BD2 domain, which prompted us to restrict the domain boundaries in our subsequent AlphaFold predictions of family-wide bromodomain homo-dimers.

**FIG. 6.**
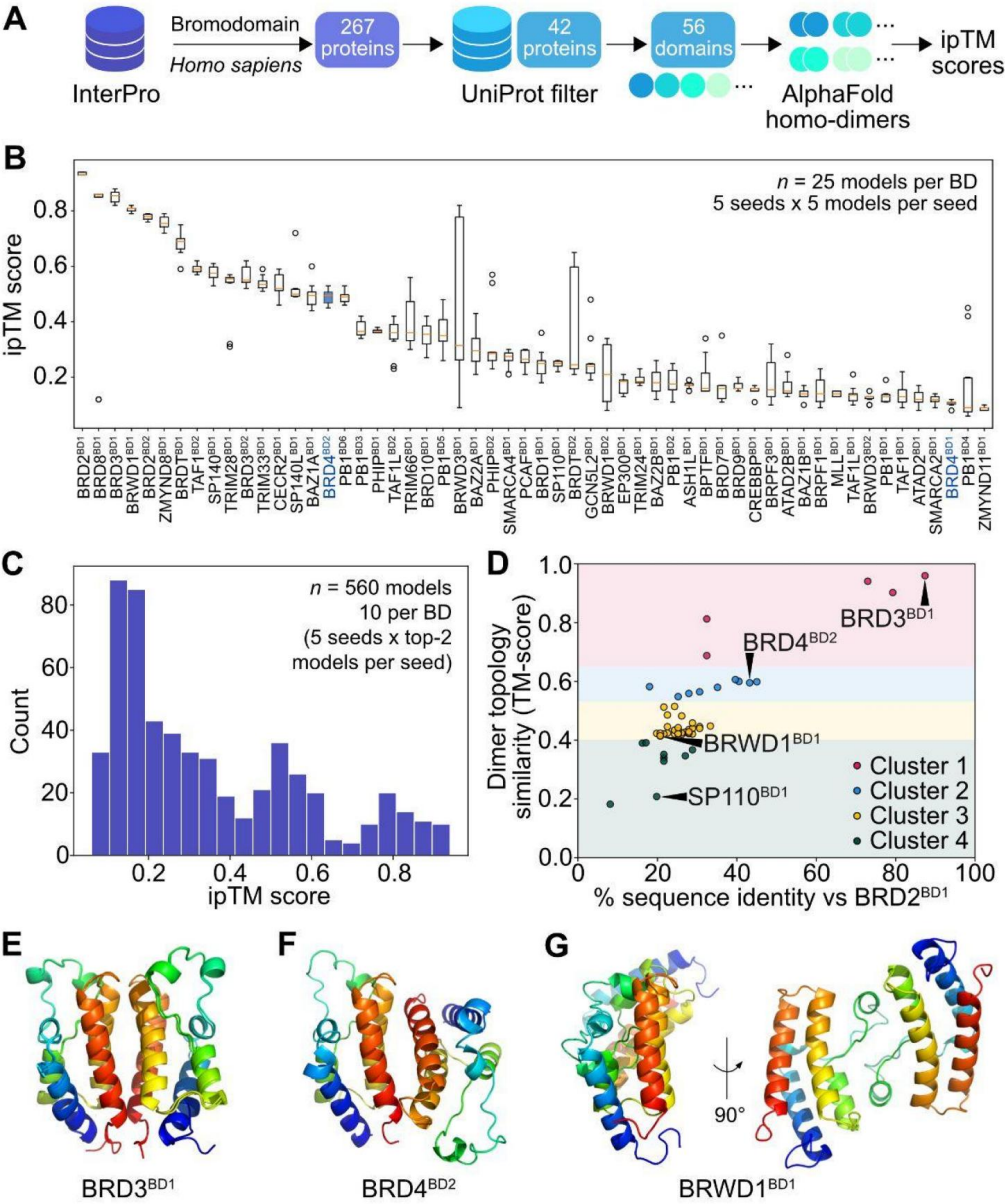
AlphaFold predictions of human bromodomain dimers. (a) A total of 56 human bromodomains were extracted from InterPro and filtered to include only those with a reviewed annotation in UniProt. Homo-dimers of each bromodomain were predicted by AlphaFold3 and AlphaFold-Multimer and ranked by ipTM scores. (b) Box plot of ipTM scores that were derived from AlphaFold3 predictions of all human bromodomains as homo-dimers. For each bromodomain, 25 ipTM scores are included (five models, five seeds). The boxes include data points that are between the first and third quartiles, the whiskers extend to 1.5-fold the interquartile range, and outliers are shown as dots. BRD4^BD2^ and BRD4^BD1^ are colored blue for reference. (c) Pooled histogram of the ipTM values from panel (a), except only the top 2-ranked models per seed are included for each bromodomain (*n* = 560 models in total). (d) Correlation of bromodomain sequence identities (x-axis) and dimer topology similarities (y-axis) with respect to BRD2^BD1^ whose dimeric structure is deposited in the PDB (1x0j). Four clusters based on dimer topology are shown in semi-transparent red, blue, yellow, and green; selected bromodomains from each cluster are labeled. Example AlphaFold3-predicted dimer models are shown for (e) BRD3^BD1^ (ipTM_max_ = 0.88), (f) BRD4^BD2^ (ipTM_max_ = 0.53), and (g) BRWD1^BD1^ (ipTM_max_ = 0.82) from the different clusters 1-3. Note that cluster 4 arises from non-canonical bromodomain folds and thus is not shown here (see supplementary material Fig. 8). In panel D, the AlphaFold3-predicted BRD3^BD1^ dimer is superimposed on the crystal structure of the BRD2^BD1^ dimer (PDB: 1x0j) to show the near-identical topology.

To our knowledge, the only reported dimers of unligated bromodomains include BRD4^BD2^ and BRD2^BD1^.^22,89^ However, more than 50 different bromodomains exist in the human proteome [Fig. 6(a)], suggesting that other dimers may exist yet have not been experimentally detected. Previous work has demonstrated that AlphaFold-Multimer can accurately predict and model the oligomeric states of proteins, including dimers.^5,21,90–93^ This is consistent with the observed similarities between the predicted BRD4^BD2^ dimeric conformation by AlphaFold3 and the second-best HADDOCK cluster. Thus, we leveraged AlphaFold-Multimer (supplementary material Table 2) and the recently developed AlphaFold3 (supplementary material Table 3) to generate structural predictions of the dimeric state for each of the 56 bromodomains in the human proteome.

For each human bromodomain, we inferred five structural models from five different random seeds, yielding a total of 25 AlphaFold predictions per bromodomain, for both AlphaFold-Multimer (supplementary material Table 2) and AlphaFold3 [Fig. 6(a), supplementary material Table 3]. In total, this sums to 2800 models of bromodomain dimers. We then visualized the distribution of ipTM scores for either all 25 models per bromodomain [Fig. 6(b)] or the top 10 models (best-2 per seed) per bromodomain [Fig. 6(c)]. A comparison of the models generated by AlphaFold-Multimer and AlphaFold3 revealed systematic differences in the prediction quality and consistency of the dimer architectures (supplementary material Fig. 11). While both methods yielded high ipTM scores for a subset of bromodomains, AlphaFold3 generated higher median ipTM scores overall and narrower distributions across the different seeds [Fig. 6(a), supplementary material Fig. 11].

We plotted the ipTM scores from all 25 models from each bromodomain [Fig. 6(b)], showing the ranges that are predicted by AlphaFold, spanning median values near 0.09 (ZMYND11^BD1^) to 0.93 (BRD2^BD1^). Approximately 27.9% (12.1%) of the top-2 ranked models per seed exhibit ipTM scores ≥0.5 (0.7), corresponding to 21 (9) out of 56 bromodomains with at least one high-confidence dimeric prediction. For BRD4^BD2^ the mean ipTM value is 0.49, which is below the AlphaFold3-defined threshold of 0.8 for a confident multimeric model. By contrast, AlphaFold3 produces very low ipTM scores near 0.10 for the N-terminal bromodomain of BRD4 (BRD4^BD1^). The latter observation agrees with NMR data, which indicate that BRD4^BD1^ is monomeric without evidence for transient dimerization.^22^ This suggests that AlphaFold3 may have discriminatory power to separate bromodomains that transiently dimerize from those that do not. Indeed, the distribution of ipTM scores among the top-2 ranked models over the five seeds (*n* = 560 models) shows that approximately one-third of the models yield ipTM scores greater than 0.4 [Fig. 6(c)].

We compared the AlphaFold3 structural predictions with and without the use of PDB templates (supplementary material Tables 3 and 4, Fig. 12, Methods). The crystal structure of the BRD2^BD1^ dimer (PDB: 1x0j) was deposited in the PDB prior to the training of both AlphaFolds, which raises the possibility that this dimeric structure may have been seen during model training. Moreover, the coordinates of the dimer may serve as a structural template when PDB templates are included. However, by removing the PDB template search from the pipeline, we tested whether the inclusion of templates impact the structural predictions. Surprisingly, the median ipTM value of the AlphaFold3 predictions increased when PDB templates were turned off (supplementary material Fig. 12).

Finally, we quantified the sequence and structural similarity among our bromodomain homo-dimers to a reference system, BRD2^BD1^. This is because a high-resolution structure of the BRD2^BD1^ dimer was previously published,^89^ and we hypothesized that sequence similarity to BRD2^BD1^ may bias the structural predictions by AlphaFold3. However, we were surprised to observe that sequence identity to BRD2^BD1^ was not correlated with ipTM scores (supplementary material Fig. 11). This suggests that AlphaFold has instead learned to identify aspects of bromodomain dimerization that are encoded within the sequences themselves, independently of templating via BRD2^BD1^. We then computed the structural similarity of all AlphaFold3 models of bromodomain dimers to BRD2^BD1^ using US-align in multimer mode.^94,95^ The resultant TM-score varies from 0 to 1, where 1 represents an identical structure while random structures return values below 0.17.^85^ Whereas ipTM was not correlated with sequence identity to BRD2^BD1^, we identified a clear correlation between the TM-score and sequence identity [Fig. 6(d)], suggesting that AlphaFold3 produces similar topological arrangements of dimers for more similar sequences.

We identified four distinct clusters of bromodomain dimer topologies. The first cluster with TM-scores near 0.7 and above contains five bromodomains whose conformations strongly resemble that of BRD2^BD1^ [Fig. 6(d)]. This is evident in the nearly identical dimeric structures of BRD2^BD1^ (PDB: 1x0j) and BRD3^BD1^ [Fig. 6(e)] in which the two protomers are arranged in a parallel manner. The next cluster with TM-scores above 0.55 contains eight bromodomain dimers in which the topological arrangement resembles our second-best HADDOCK cluster of BRD4^BD2^ [Figs. 4(b), 4(c), 5, and 6(f)], wherein one protomer has been rotated nearly 90° in orientation. The third and most populous cluster with TM-scores between 0.4 and 0.55 contains dimers in which the interface varies from more cluster 2-like to a side-by-side arrangement involving the ZA and BC loops at the interface [Fig. 6(g)]. The fourth cluster contains further distinct dimer interfaces (supplementary material Fig. 11), and it is unclear if the latter orientations are physically meaningful.

Finally, we examined if AlphaFold3 predicts the formation of bromodomain hetero-dimers. To this end, we restricted our predictions to the BET-family proteins that harbor tandem BD1 and BD2 domains: BRD2, BRD3, BRD4, and BRDT. AlphaFold3 generally tends to predict higher ipTM scores for bromodomain hetero-dimers involving the same domain type, i.e., BD1-BD1 and BD2-BD2 score higher than BD1-BD2 hetero-dimers (supplementary material Table 5). For example, the BRD4^BD2^-BRD2^BD2^ and BRD2^BD1^-BRD3^BD1^ hetero-dimers yield ipTM scores of 0.76 and 0.91, respectively. However, a notable exception to this trend is for BRD4, where the BRD4^BD1^-BRD4^BD2^ hetero-dimer produces an ipTM score of 0.53, which is identical to the top-scoring prediction of the BRD4^BD2^ homo-dimer (ipTM_max_ 0.53).

Overall, our AlphaFold-based screen of human bromodomain dimers suggests that other metrics than simply sequence identity to BRD2^BD1^ or PDB templating affect the ipTM scores. This indicates that bromodomain dimerization may be encoded by co-evolving residues within the multiple sequence alignments of bromodomains. The relatively moderate ipTM score of 0.49 for BRD4^BD2^, as opposed to 0.1 for BRD4^BD1^, for which NMR evidence suggests it remains monomeric, indicates that AlphaFold may be able to identify transient bromodomain dimers. Further, there are 15 other bromodomains with similar or higher mean ipTM scores than BRD4^BD2^, suggesting that bromodomain dimerization may be more widespread than appreciated.

## DISCUSSION

Bromodomains are a conserved class of reader domains that recognize acetylated lysine residues on histones and other proteins involved in transcription and chromatin recognition.^31,32^ Pharmacological inhibition of BET proteins has widespread effects in numerous disease models,^96^ particularly in cancer, inflammatory diseases, and metabolic disorders, underscoring their functional importance in transcriptional control.^97^ Generally, bromodomains are presumed to function as monomeric units. However, the dynamic nature of protein oligomerization can render quaternary-structure determination and annotation challenging, especially when self-assembly is weak and transient. Homo-dimerization has been reported for both BRD2^BD1^^89^ and BRD4^BD2^^22,40^ as well as for full-length BRD4.^98^ Other bromodomains, such as those in GCN5 and PCAF form dimers in the crystal lattice that were not detected in solution.^31^ Despite seemingly weak dimerization propensity, bromodomain dimerization that is transient under dilute conditions may become functionally relevant within condensates where bromodomain concentrations are highly enriched.^36,39^

Here, we characterized the transient dimerization of BRD4^BD2^ by a range of NMR methods, including CSPs, 2D lineshape analysis, and ^15^N-CPMG relaxation dispersion to detect residues that are involved in the dimer interface. In addition, we probed the hydrodynamic properties of BRD4^BD2^ with ^15^N spin relaxation, PFG-NMR translational diffusion, and SAXS measurements. Our NMR data indicate that the BRD4^BD2^ dimer has a transient lifetime of approximately 1 ms and a *K*_d_ near 400 μM. Based on these values, BRD4^BD2^ would be primarily monomeric *in vivo* where its concentration is expected to be in the nanomolar range. However, inside BRD4-containing condensates that include other components of transcriptional machinery, the effective BRD4^BD2^ concentration may be sufficiently high so as to promote dimer formation. Indeed, BRD4 phase separation depends both on its intrinsically disordered regions and its tandem bromodomains,^37^ indicating that the multivalent interactions involving bromodomains are required for condensation. In such condensates, BRD4^BD2^ is additionally expected to be exposed to a high local effective concentration of acetylated lysine residues. Previous NMR measurements indicated that acetyl-lysine peptide binding to BRD4^BD2^ did not alter its dimerization propensity,^22^ which suggests that dimerization of condensate-localized BRD4^BD2^ may cluster together different acetyl-lysine-bound BRD4 molecules.

Using NMR spectroscopy, we identified the BRD4^BD2^ residues that are involved in dimer formation and generated an NMR data-driven model of the BRD4^BD2^ dimer. The interfacial residues are largely polar and charged, with two histidine residues (H396, H437) whose protonation state may influence dimerization.^99^ The overall topology of our HADDOCK-derived BRD4^BD2^ model differs from the only other known structure of an unligated BD dimer (BRD2^BD1^; TM-score = 0.6) due to the rotation of one protomer relative to another. We observed that the dimer topology in the second-best HADDOCK cluster matches that predicted by AlphaFold3 with an average ipTM score of 0.49. This prompted us to explore predicted dimerization propensity across the bromodomain family. We used AlphaFold-Multimer and AlphaFold3 to predict dimeric complexes for all BDs in the human proteome and observed a range of different dimers that could be clustered into four distinct groups. As compared to BRD4^BD2^, for which experimental evidence confirms its dimerization propensity, there are 15 other BDs with higher median ipTM scores among the top-2 models across five seeds. Only one of these bromodomains has previously been reported to dimerize (BRD2^BD1^). Overall, there are 21 BDs that have at least one AlphaFold prediction in which the dimer has an ipTM score that is greater than or equal to BRD4^BD2^.

Moreover, AlphaFold3 may not be able to accurately identify some transient homo-dimers: for instance, the ATAD2 bromodomain (128 residues) yielded a rotational correlation time of 12.7 ns at 298 K,^100^ suggesting transient dimerization, although our AlphaFold3 predictions yield low ipTM scores near 0.1 for the ATAD2 dimer. Thus, without knowledge of the false negative and false positive rates for AlphaFold3 predictions of homo-dimers, our combined NMR and AlphaFold results suggest that transient dimerization may be more widespread across the BD superfamily. In this context, NMR spectroscopy is well suited to detect and characterize weak dimerization in solution. Bromodomains are near 13-15 kDa as monomers and thus the dimeric states (26-30 kDa) remain accessible to conventional NMR methods, as shown here. The spectral quality would be further improved by selective deuteration^101^ or perdeuteration^102^ followed by back-protonation at the amide positions, to reduce dipolar relaxation from nearby aliphatic protons, as shown previously for a tandem BRD4^BD1,BD2^ construct.^22^

What differentiates the capacity to dimerize between different bromodomains? One or several mutations on protein surfaces can readily create or disrupt homo-oligomeric interfaces,^103^ because their impacts are multiplied by symmetry.^104,105^ In the absence of selective pressures on a particular oligomeric state, new quaternary structures can rapidly evolve within protein families.^106–108^ This reflects the relatively high mutability of protein surfaces,^109,110^ as well as the small compositional differences between solvent-exposed patches and protein-protein interfaces.^111^ Indeed, single mutations in small monomeric domains with no evidence for dimerization produced dimers that formed with micromolar dissociation constants.^54,112,113^ An additional two mutations yielded a dimer with a sub-nanomolar dissociation constant.^114^ Thus, quaternary structure is highly evolvable and new oligomeric forms can emerge from only one or several sequence changes. In the case of bromodomains, it will be interesting to explore the evolutionary relationships between their sequences and oligomeric states, including the possibility for hetero-dimer formation, in more depth.

## METHODS

### Protein expression and purification

DNA encoding for human BRD4^BD2^ (UniProt ID: O60885, residues E345-E460) was synthesized by GenScript and codon-optimized for expression in *Escherichia coli*. Prior to the first BRD4^BD2^ residue, the synthesized gene included a hexahistidine tag (His_6_) followed by a SUMO tag. The codon-optimized DNA was then sub-cloned into a pET-29b(+)-derived expression vector (GenScript). The total length of the construct is 116 residues and the molecular mass is 13.5 kDa.

For protein expression, *E. coli* BL21(DE3) cells were transformed with the BRD4^BD2^ expression plasmid by heat shock and plated on LB agar containing 50 μg/mL kanamycin. Single colonies were used to inoculate overnight precultures grown in LB medium containing 50 μg/mL kanamycin at 37 °C with shaking. Large-scale cultures were inoculated to an initial OD_600_ of approximately 0.1 and grown at 37 °C until OD_600_ reached 0.4-0.6. Protein expression was induced by addition of isopropyl *β*-D-1-thiogalactopyranoside (IPTG) to a final concentration of 0.25 mM, followed by overnight expression at 18 °C. Cells were harvested by centrifugation at 4 °C and resuspended in lysis buffer (50 mM Tris-HCl, 500 mM NaCl, pH 8.0) supplemented with DNase I, MgSO_4_, protease inhibitor cocktail, and lysozyme. Cell lysis was performed using sonication. The lysate was clarified by high-speed centrifugation, and the supernatant was applied to Ni^2+^-nitrilotriacetic acid (Ni-NTA) affinity resin that was pre-equilibrated in lysis buffer. After extensive washing with lysis buffer, His_6_-SUMO-BRD4^BD2^ was eluted with lysis buffer that was supplemented with 500 mM imidazole. The His_6_-SUMO tag was removed by overnight digestion with His_6_-tagged SUMO protease (Ulp1) during dialysis against lysis buffer at 4 °C. Cleaved BRD4^BD2^ was then separated from the His_6_-tagged SUMO and SUMO protease by reverse Ni-NTA affinity chromatography. The flow-through contained untagged BRD4^BD2^, which was subsequently buffer-exchanged and concentrated using centrifugal ultrafiltration (Amicon, 3 kDa molecular-weight cutoff) into NMR buffer (25 mM MES, 50 mM NaCl, 0.5 mM EDTA, 1 mM TCEP, 0.02% sodium azide, pH 6.5).

For NMR experiments that required isotope-labelled protein, precultures were harvested by centrifugation and transferred into M9 minimal medium supplemented with ^15^NH_4_Cl as the sole nitrogen source and natural-abundance glucose (^15^N-labelled samples). For uniformly ^13^C, ^15^N-labeled samples, the cells were grown in ^13^C, ^15^N-labeled rich growth media OD2 for *E. coli* (Silantes Gmbh, Munich, Germany). Protein expression and purification then proceeded as described above.

Protein purity was assessed by sodium dodecyl sulfate-polyacrylamide gel electrophoresis (SDS-PAGE), and correct folding was verified by circular dichroism spectroscopy. Final protein concentrations were determined by UV absorbance at 280 nm using an extinction coefficient of 14 440 M^−1^ cm^−1^. Samples were stored at 4 °C for short-term use, while aliquots for long-term storage were flash-frozen in liquid nitrogen and stored at −80 °C.

### NMR spectroscopy

NMR samples contained ^15^N-labeled BRD4^BD2^ in 25 mM MES, 50 mM NaCl, 0.5 mM EDTA, 1 mM TCEP, and 0.02% (w/v) sodium azide at pH 6.5 with 5% (v/v) D_2_O added for the lock signal. Unless stated otherwise, spectra were recorded at 293 K on Bruker Avance III spectrometers operating at 500 or 600 MHz (^1^H frequency) equipped with cryogenically cooled, triple-resonance probeheads with a single z-axis gradient. All NMR spectra were acquired using TopSpin v3.8.0, processed with NMRPipe,^115^ and visualized with NMRFAM-Sparky.^116^

### Resonance assignments

Backbone and Cβ resonance assignments were obtained using standard triple-resonance experiments recorded on a sample of ^13^C,^15^N-labelled BRD4^BD2^. Three-dimensional spectra were collected with non-uniform sampling (NUS) using random sampling schedules and the following sparsity levels: HNCA (20%), HN(CO)CA (20%), HNCACB (25%), HNCO (20%), HN(CA)CO (35%). The 3D spectra were reconstructed using SMILE,^117^ processed using NMRPipe,^115^ and analyzed using NMRFAM-Sparky.^116^ The 3D spectra were recorded at 500 MHz ^1^H Larmor frequency at 310 K using a protein concentration of 200 μM. This temperature and protein concentration were chosen to (1) decrease the rotational correlation time and thus increase *T*_2_ times and (2) reduce the molar fraction of dimer (ca. 39% dimer given *K*_d_ = 391 μM) and thereby increase the fraction of molecules with *T*_2_ times of the monomer, while also enabling sufficient signal-to-noise in the 3D assignment spectra. The assigned 2D ^1^H-^15^N HSQC spectrum is shown in supplementary material Fig. 1. To enable assignment transfer from 310 K to the lower temperatures that were used in subsequent experiments, a temperature titration series of 2D ^1^H-^15^N HSQC spectra was recorded at 293, 298, 303, 308, and 310 K (supplementary material Fig. 1). ^1^H chemical shifts were referenced internally to DSS (4,4-dimethyl-4-silapentane-1-sulfonic acid) and the ^13^C and ^15^N chemical shifts were referenced indirectly following standard procedures.^118^ The referenced chemical shifts (^1^H^N^, ^15^N, ^13^CO, ^13^CA, ^13^CB) were supplied to the TALOS-N^44^ webserver (https://spin.niddk.nih.gov/bax/nmrserver/talosn/) with identical sequences excluded, automated offset correction, and ^1^H shifts included (supplementary material Fig. 2).

The number of assigned resonances was compared against the total number of expected resonances based on the amino-acid sequence, which is 116 residues in length. For ^1^H^N^ and ^15^N chemical shifts, the N-terminus and all six Pro residues were removed, yielding a total of 109 possible signals. For ^13^CA and ^13^CO chemical shifts, only P434 was removed because it lies directly adjacent to P435 and thus it cannot be observed in our ^1^H^N^-detected experiments, yielding a total of 115 possible signals. For ^13^CB chemical shifts, P434 was removed as well as three Gly residues, yielding a total of 112 possible signals.

### Chemical shift perturbation (CSP) analysis

Weighted, combined chemical shift perturbations (CSPs) are reported for the comparison of 2D ^1^H-^15^N HSQC spectra recorded at 500 MHz and 293 K at BRD4^BD2^ concentrations of 9 and 910 μM. CSPs were calculated using the standard weighted chemical shift difference:

$$\Delta \delta =\sqrt{{\left(\Delta \delta_{H}\right)}^{2}+{\left(0.154\cdot \Delta \delta_{N}\right)}^{2}},$$ (1)

where Δδ_H_ and Δδ_N_ are the differences in ^1^H and ^15^N chemical shifts, respectively, and the weighting factor for the ^15^N dimension (0.154) reflects the larger chemical shift range for backbone amide nitrogens. Residues exhibiting CSP values greater than one standard deviation (SD) above the mean were classified as significantly perturbed and selected for subsequent analysis.

### 2D NMR lineshape analysis

2D ^1^H-^15^N-HSQC spectra were recorded at 500 MHz at 293 K with ^1^H, ^15^N spectral widths and acquisition times of (8012, 1014 Hz) and (64, 63 ms), respectively. Solvent suppression was achieved with a WATERGATE element^119^ that included water flip-back pulses to reduce signal loss from exchangeable protons.^120^ Water-selective 90° pulses were applied with the shape defined by the central lobe of a sinc function. The inter-scan delay was set to 1 s and ^15^N decoupling during ^1^H acquisition was achieved with a 1.25-kHz WALTZ-16 field.^121^ The total BRD4^BD2^ concentration was varied, with the acquisition times and receiver gain fixed while the number of scans was adjusted depending on protein concentration. The values of protein concentration and the number of scans were as follows (total BRD4^BD2^ in μM, number of scans): (9.1, 440), (25, 400), (91, 32), (250, 16), (500, 4), and (911, 4).

The concentration-dependent 2D ^1^H-^15^N-HSQC spectra of ^15^N-labeled BRD4^BD2^ were analyzed using the software NMR-TITAN^45^ to extract kinetic and thermodynamic parameters of the monomer-dimer equilibrium. NMR-TITAN was used within the software framework of the SBGrid Consortium.^122^ All 2D ^1^H-^15^N HSQC spectra were collected without a sensitivity enhancement scheme and processed identically using exponential window functions (4-Hz line broadening), as specified by NMR-TITAN.

A global two-state exchange model (monomer-dimer) was used to simultaneously fit 18 resonances with discernible CSPs across the concentration series. These resonances included: E352, G386, T402, F426, S427, G419, R423, N433, H437, V440, A441, Q447, V449, E451, F454, M457, Q-NH_2_, and unassigned (likely V439, based on BMRB 51417), where Q-NH_2_ refers to a Gln side-chain that was not assigned. Given a known total protein concentration (*P*_t_) and dimer dissociation constant (*K*_d_), the molar fraction of monomer is obtained through mass balance and described by the following equations:

$$K_{d}=\frac{{\left[M\right]}^{2}}{\left[D\right]},$$ (2)

$$P_{t}=\left[M\right]+2\left[D\right],$$ (3)

$$\frac{2{\left[M\right]}^{2}}{K_{d}}+\left[M\right]-P_{t}=0,$$ (4)

$$\left[M\right]=\frac{K_{d}}{4}\left(\sqrt{1+\frac{8P_{t}}{K_{d}}}-1\right),$$ (5)

by solving the quadratic equation (4) for [M], yielding Eq. (5) as the physically meaningful root.

In NMR-TITAN, partially overlapped signals were fit jointly as groups. Both protomers within the dimer (D1, D2) were treated identically because there was no NMR evidence for dimer asymmetry, and thus D1 and D2 shared all fitted parameters (i.e., ω_H,D1_ = ω_H,D2_; ω_N,D1_ = ω_N,D2_; R_2H,D1_ = R_2H,D2_; R_2N,D1_ = R_2N,D2_). Exchange parameters (*k*_off_, *K*_d_) were optimized globally, while the ^1^H and ^15^N chemical shifts and transverse ^1^H and ^15^N relaxation rates for the monomer (M) and dimer (D1 = D2) were allowed to vary in a site-specific manner. The reported parameters represent best-fit values obtained from a global analysis based on 100 bootstrapped fits: *K*_d_ = 391 ± 12 μM, *k*_off_ = 944 ± 65 s^−1^, and *k*_on_ = 2.42 ± 0.18 × 10^6^ M^−1^ s^−1^.

### ^15^N relaxation analysis and estimation of rotational correlation times

^15^N longitudinal (*T*_1_) and rotating-frame (*T*_1ρ_) spin relaxation measurements were recorded at 298 K at a static magnetic field strength of 14.1 T (600 MHz ^1^H Larmor) using established pulse sequences that include water flip-back and a sensitivity-enhanced detection scheme^123^ as described by Lakomek *et al.*^67,68^ BRD4^BD2^ samples were prepared at protein concentrations of 150 μM, 1 mM, and 1.6 mM with either ^15^N (0.15 mM) or ^13^C,^15^N-labeled samples (1, 1.6 mM). The ^1^H, ^15^N spectral widths and acquisition times were (9615, 1156 Hz) and (64, 69 ms), respectively. In both experiments, the inter-scan delay was set to 2.5 s during which ^15^N temperature-compensation pulses^124^ were applied 50-kHz off-resonance. The number of scans was either eight (1, 1.6 mM BRD4^BD2^) or 16 (0.15 mM).

Cross-correlated relaxation between ^15^N chemical shift anisotropy and ^1^H-^15^N dipole-dipole interactions was refocused with amide-proton selective inversion pulses using an IBURP-2 shape.^125^ The duration of the IBURP-2 pulse was 2 ms (at 600 MHz), and it was centered at an offset of 8.67 ppm. The ^15^N spin lock during the *T*_1ρ_ was applied with a *B*_1_ field of 2 kHz, with the adiabatic half passages matched to the same field strength.

For ^13^C,^15^N-labeled samples, ^13^C spins were inverted to refocus any cross-correlated relaxation between ^13^C and ^15^N. Selective inversion was achieved with 180° pulses of rectangular shape with the duration defined by $\sqrt{3}/(2\Delta \upsilon )$^126^ where Δυ refers to the difference between the centers of the ^13^CA and ^13^CO chemical shift ranges at 600 MHz (ca. 18.1 kHz), corresponding to a 180° pulse duration of 47.8 μs with the power level adjusted accordingly.

Peak intensities from 2D ^1^H-^15^N HSQC correlation spectra recorded with variable relaxation delays were quantified using PeakFit (version 0.10.5; https://github.com/gbouvignies/PeakFit). Relaxation delay times were computed from the corresponding variable counter (vc) and variable pulse (vp) lists that were used during acquisition. Relaxation times were determined by fitting the peak intensities to a monoexponential decay, and relaxation rates were calculated as *R*_1_ = 1/*T*_1_ and *R*_2_ = 1/*T*_2_. For both the *T*_1_ and *T*_1ρ_ experiments, 19 well-resolved cross-peaks were selected for analysis, excluding resonances that were located in regions exhibiting elevated flexibility as evidenced by decreased *R*_2_ rates. The overall analysis strategy followed established relaxation analysis procedures.^63^ *R*_1ρ_ relaxation rates were converted to transverse relaxation rates (*R*_2_) by correcting for ^15^N off-resonance effects and the ^15^N spin-lock field strength according to Eq. (6):

$$R_{2}=\frac{R_{1\rho }}{\text{si}n^{2}\theta }-\frac{R_{1}}{\text{ta}n^{2}\theta };\text{ tan }\theta =\frac{\omega_{1}}{\Omega },$$ (6)

where $\omega_{1}$ is the spin-lock field strength and Ω the ^15^N resonance offset. For each protein concentration, the rotational correlation time (*τ_c_*) was estimated assuming isotropic tumbling, using the analytical expression based on the *T*_1_/*T*_2_ ratio and the ^15^N Larmor frequency [Eq. (7)]. The reported *τ_c_* values at each protein concentration represent the mean ± standard deviation (SD) across the analyzed residues (*n* = 19 per concentration)

$$\tau_{c}\approx \frac{1}{4{\pi v}_{N}}\sqrt{6\frac{T_{1}}{T_{2}}-7},$$ (7)

where $v_{N}$ is the ^15^N Larmor frequency at 14.1 T (60.838 MHz). To provide reference values for the expected tumbling of pure BRD4^BD2^ monomer and pure BRD4^BD2^ dimer, theoretical τ_c_ values were calculated with HydroNMR^69^ derived rotational diffusion tensors. We used as input a PDB structure of the BRD4^BD2^ monomer (PDB: 2ouo) and an AlphaFold3-generated model of a synthetic BRD4^BD2^ dimer. Assuming isotropic tumbling, τ_c_ was computed from the 3 × 3 rotational diffusion tensor D_rr_ as

$$\tau_{c}=\frac{1}{6Tr{(D}_{rr})},$$ (8)

where *Tr*(D_rr_) refers to the trace of the *D*_rr_ matrix. The resulting monomer and dimer τ_c_ values were used as grey reference bars in Fig. 3.

### ^15^N-CPMG relaxation dispersion

^15^N-CPMG relaxation dispersion data were collected at 500 MHz and 293 K using a 0.91 mM sample of ^15^N-labeled BRD4^BD2^. We used a modified form of the ^15^N ST-CW-CPMG pulse sequence,^52,59^ which includes ^1^H continuous-wave decoupling to maintain in-phase ^15^N magnetization during the CPMG pulse train^51^ and [0013] phase cycling of the ^15^N CPMG pulse that was previously shown to reduce offset-dependent artifacts.^127^ The data were recorded in pseudo-3D format in which the number of ^15^N CPMG pulses varied across 22 planes with two duplicated planes for error analysis. The reference plane excluded the relaxation delay (*T*_relax_) of 40 ms, whereas the other 21 planes included *T*_relax_ with v_CPMG_ values ranging from 25 to 1000 Hz. During *T*_relax_, ^1^H continuous-wave decoupling and the ^15^N CPMG pulse were applied using *B*_1_ fields of 15.6 and 5.6 kHz, respectively. The values of v_CPMG_ are defined as v_CPMG_ = 1/2 δ, where δ is defined as the time between the successive centers of the ^15^N CPMG pulses, given by δ=2τ_CP_ + 2pw_CPMG_, where 2pw_CPMG_ is the pulse width of the 180° ^15^N CPMG pulse (90 μs). For example, at v_CPMG_ = 1000 Hz, δ then equals 0.5 ms and thus τ_CP_ is equal to 0.41 ms. The inter-scan delay time was set to 3 s and the ^1^H and ^15^N acquisition times were 64 and 63 ms, respectively. ^15^N decoupling during ^1^H acquisition was achieved using a WALTZ-16^121^ field of 1.2 kHz, and ^15^N temperature-compensation pulses (50 kHz off-resonance)^124^ were applied during the inter-scan delay for a duration that depended inversely on the number of ^15^N CPMG pulses.

Relative peak intensities were determined by lineshape fitting with FuDA.^128^ The effective ^15^N *R*_2_ (*R*_2,eff_) was then calculated by

$$R_{2,\text{eff}}=\frac{-\text{ln }\left(\frac{I_{j}}{I_{0}}\right)}{T_{\text{relax}}},$$ (9)

where *I*_0_ corresponds to the peak intensity in the reference plane (no *T*_relax_), *I*_j_ to the peak intensity in plane *j* with the value v_CPMG,j_, and *T*_relax_ to the 40-ms relaxation delay time. Residues with *R*_ex_ = *R*_2,eff_ (25 Hz) − *R*_2,eff_ (1000 Hz) > 2 s^−1^ were globally fit to a two-state chemical exchange model in *ChemEx* (https://github.com/gbouvignies/ChemEx) described by the global parameters *k*_ex_ and *p*_B_ and site-specific parameters ^15^N-*R*_2A_ and Δω_N_. Under the conditions where we acquired ^15^N-CPMG relaxation dispersion data, state B corresponds to the BRD4^BD2^ monomer and state A to the BRD4^BD2^ dimer, with the interconversion rate *k*_ex_ described by:

$$k_{ex}=k_{\text{off}}+{2k}_{on}[M].$$ (10)

The rate constants *k*_off_ and *k*_on_ can then be directly calculated based on the values of *k*_ex_ and *p*_B_ with the following equations:

$$k_{\text{off}}=k_{ex}p_{B},$$ (11)

$$k_{on}=\frac{k_{ex}(1-p_{B})}{2p_{B}P_{T}}.$$ (12)

Although global fitting of CPMG relaxation dispersion data obtained at two static magnetic field strengths dramatically reduces parameter inter-dependencies,^129^ we only obtained data at a single static magnetic field strength. To reduce parameter correlations while fitting single-field CPMG relaxation dispersion data, we fixed the *p*_B_ based on our NMR-TITAN-derived *K*_d_ value and the known total BRD4^BD2^ protein concentration. All other exchange parameters were allowed to float in the fit.

### Translational diffusion measurements

PFG translational diffusion experiments were recorded at 600 MHz and 298 K at protein concentrations of 15, 150 μM, 1.0, and 1.6 mM in a standard 5-mm NMR tube. We used water-suppressed bipolar pulse-pair longitudinal encode-decode (BPP-LED) pulse sequences to record either ^1^H or ^15^N-edited ^1^H diffusion spectra^130–132^ as pseudo-2D datasets, in which the encoding and decoding gradient strengths were varied. ^15^N-edited ^1^H diffusion experiments were used to selectively monitor the backbone amide resonances of BRD4^BD2^. Complementary ^1^H diffusion experiments were recorded to observe buffer signals and account for viscosity effects. Diffusion encoding was implemented using a linear gradient ramp comprising 12 gradient strengths between 2% and 98% of the maximum gradient strength. The diffusion delay Δ was set to 200 ms, and the gradient pulse duration *δ* was 1 ms.

All datasets were processed using NMRPipe and integrals were quantified using custom Python scripts based on the *nmrglue* library^133^ and modified from Ref. 134. For ^15^N-edited experiments, signal intensities were obtained by integrating a defined amide proton chemical-shift window (6.0-10.0 ppm) for each gradient increment. For buffer diffusion experiments, a well-resolved ^1^H resonance (3.79-3.86 ppm) from EDTA was integrated. Intensities were normalized to the weakest gradient strength (I_0_).

Diffusion attenuation was analyzed using the linearized Stejskal-Tanner equation by fitting ln(I/I_0_) as a function of the squared gradient strength. Linear least-squares fits constrained to pass through the origin were applied. Because all diffusion experiments were recorded under the same conditions with identical NMR parameters, the relative diffusion properties could be directly compared without conversion to absolute diffusion coefficients. Uncertainties of the fitted slopes were estimated using a leave-one-out bootstrap procedure in which the fit was repeated after randomly omitting one gradient point in each iteration, and the standard deviation of the resulting slope distribution was taken as the error.

### SAXS

SAXS experiments were performed on an in-house SAXS instrument (Rigaku BioSAXS-1000) attached to a Rigaku HF007 microfocus rotating anode generator with a copper target (40 kV, 30 mA), using the Cu Kα wavelength (1.54 Å). Transmissions were measured with a photodiode beam stop. The scattering vector calibration was done with a silver behenate sample. The samples were loaded with an automated 96-well sample changer. Sample temperature was controlled using a Julabo F25-MA thermostat with a specified temperature stability of ±0.02 K. Measurements consisted of eight 900 s frames, which were compared to check for radiation damage before averaging, for a total measurement duration of 7200 s per sample. BRD4^BD2^ samples were prepared at protein concentrations of 100, 300, 500, and 1000 μM in the same buffer used for NMR experiments (25 mM MES, 50 mM NaCl, 0.5 mM EDTA, 1 mM TCEP, 0.02% sodium azide, pH 6.5). Measurements were performed at 293 K to match the conditions under which the *K*_d_ was determined by NMR spectroscopy. Corresponding buffer measurements were collected under identical conditions and subtracted from the sample scattering profiles prior to analysis. Further, we identified some protein aggregation in the 1000 μM sample as evidenced by an uptick in the low *q* range. Circular averaging of raw images, averaging of frames and solvent subtraction were performed using the Rigaku SAXSLab software (version 3.1.1). The *PRIMUS* software suite was used for all subsequent analysis steps.^79^

### HADDOCK calculations

To identify residues that exhibit CSPs and are solvent-exposed (and thus suitable to use as docking restraints), the relative solvent accessibility (RSA) was calculated from the structure of the BRD4^BD2^ monomer (PDB: 2ouo) using FreeSASA.^135^ Residues with RSA values greater than or equal to 15% were classified as solvent-accessible and retained as active residues for docking, while residues below this threshold were excluded. Integrative docking calculations were performed using the HADDOCK2.4 web server.^82,136^ Solvent-accessible residues with CSPs were defined as active residues, while passive residues were automatically assigned by HADDOCK based on spatial proximity. Docking was performed assuming a homodimeric complex using default HADDOCK2.4 parameters.

A total of 200 initial models were generated, followed by semi-flexible refinement and final refinement in explicit solvent. Clustering was performed based on RMSD, and clusters were ranked according to the HADDOCK score. In total, 156 water-refined models were clustered into seven clusters, representing 78% of the final models. The HADDOCK results were evaluated based on cluster size, HADDOCK score, RMSD, and energetic contributions including van der Waals, electrostatic, desolvation, and restraint-violation energies. The top-ranking cluster exhibited the most favorable HADDOCK score and Z-score and was selected for further analysis. The interfacial area of the HADDOCK model was computed with the PDBePISA webserver.^137^ The total buried surface area is reported, i.e., from both protomers. We considered the interfacial residues as well as the buried surface area contributed by each interfacial residue. By residue type, the residues at the interface comprised 30% polar (Q353, H396, Q416, S427, N428, Y430, Y432, N433, Q447), 37% hydrophobic (I393, I394, A420, L424, P434, P435, M452, F454, A455, M457, P458), and 33% charged (K395, D421, R423, K431, D436, R444, D448, E451, K456, D459).

### Bromodomain dimer structure prediction using AlphaFold3 and AlphaFold-multimer (ColabFold)

Human bromodomain-containing proteins were identified from the Swiss-Prot-reviewed UniProt human reference proteome (UP000005640), taxonomy accession 9605, based on annotation with the InterPro bromodomain entry (IPR001487). A total of 56 bromodomains were used from 42 different proteins. Bromodomain residue boundaries were defined using UniProt and InterPro domain annotations.

Structure predictions of bromodomain dimers were generated using AlphaFold-Multimer (ColabFold) and AlphaFold3. Predictions were performed using default ColabFold settings for the model alphafold2_multimer_v3 (20 recycles, 5 models) and the same values were used in the AlphaFold3 predictions. We generated 5 random seeds for each bromodomain, yielding a total of 25 inferred structural models per bromodomain. The random seeds for the AlphaFold3 models were 10, 55, 99, 424, and 777. For dimeric models, interface quality was assessed using the interface predicted template modelling (ipTM) score and for structural clustering, the top-ranked dimeric models (as reported by ColabFold and AlphaFold3) were selected for analysis. For AlphaFold3, which produces mmCIF-format structures, the structures were converted to PDB format for US-align (see below). The predicted structural models of human bromodomain homo-dimers will be deposited in the Model Archive.^138^

### Sequence and structural comparisons among bromodomains

Pairwise sequence comparisons were performed using *Biopython*.^139^ All bromodomain sequences were globally aligned to the BRD2^BD1^ bromodomain sequence, which was used as a reference. Sequence identity was calculated as the number of aligned residues divided by the length of the BRD2^BD1^ sequence. To compare the predicted dimer topologies, structural alignments were performed using US-align^94,95^ in multimer mode. The experimentally determined BRD2^BD1^ dimer structure (PDB ID: 1x0j) was used as the reference structure. Each predicted bromodomain dimer was aligned to the BRD2^BD1^ reference dimer, and similarity was quantified using the TM-score which ranges between 0 and 1. Random structures typically score near or below 0.2,^85^ whereas values above 0.5 generally indicate the same fold (monomer) or topology (oligomer).^95^

## SUPPLEMENTARY MATERIAL

See the supplementary material for additional figures and tables related to the results described in the main text.

## Data Availability

The data that support the findings of this study are openly available in GitHub at https://github.com/alisadengler/bromodomain-dimers-data, Ref. 140.
